# A Novel CMY Variant Confers Transferable High-Level Resistance to Ceftazidime-Avibactam in Multidrug-Resistant Escherichia coli

**DOI:** 10.1128/spectrum.03349-22

**Published:** 2023-02-14

**Authors:** Junxin Zhou, Weiping Wang, Min Liang, Qian Yu, Shiqi Cai, Tailong Lei, Yan Jiang, Xiaoxing Du, Zhihui Zhou, Yunsong Yu

**Affiliations:** a Department of Infectious Diseases, Sir Run Run Shaw Hospital, Zhejiang University School of Medicine, Hangzhou, China; b Key Laboratory of Microbial Technology and Bioinformatics of Zhejiang Province, Hangzhou, China; c Regional Medical Center for National Institute of Respiratory Diseases, Sir Run Run Shaw Hospital, Zhejiang University School of Medicine, Hangzhou, China; d Department of Clinical Laboratory, Jinling Hospital, Medical School of Nanjing University, Nanjing, China; Yangzhou University

**Keywords:** *Escherichia coli*, *bla*_CMY-178_, ceftazidime-avibactam resistance, IncI1 plasmid

## Abstract

Here, our objective was to explore the molecular mechanism underlying ceftazidime-avibactam resistance in a novel CMY-178 variant produced by the clinical Escherichia coli strain AR13438. The antibiotic susceptibility of the clinical isolate, its transconjugants, and its transformants harboring transferable *bla*_CMY_ were determined by the agar dilution method. S1-PFGE, cloning experiments, and whole-genome sequencing (WGS) were performed to investigate the molecular characteristics of ceftazidime-avibactam resistance genes. Kinetic parameters were compared among the purified CMY variants. Structural modeling and molecular docking were performed to assess the affinity between the CMYs and drugs. The horizontal transferability of the plasmid was evaluated by a conjugation experiment. The fitness cost of the plasmid was analyzed by determining the maximal growth rate, the maximum optical density at 600 nm (OD_600_), and the duration of the lag phase. AR13438, a sequence type 457 E. coli strain, was resistant to multiple cephalosporins, piperacillin-tazobactam, and ceftazidime-avibactam at high levels and was susceptible to carbapenems. WGS and cloning experiments indicated that a novel CMY gene, *bla*_CMY-178_, was responsible for ceftazidime-avibactam resistance. Compared with the closely related CMY-172, CMY-178 had a nonsynonymous amino acid substitution at position 70 (Asn70Thr). CMY-178 increased the MICs of multiple cephalosporins and ceftazidime-avibactam compared with CMY-172. The kinetic constant *K_i_* values of CMY-172 and CMY-178 against tazobactam were 2.12 ± 0.34 and 2.49 ± 0.51 μM, respectively. Structural modeling and molecular docking indicated a narrowing of the CMY-178 ligand-binding pocket and its entrance and a stronger positive charge at the pocket entrance compared with those observed with CMY-172. *bla*_CMY-178_ was located in a 96.9-kb IncI1-type plasmid, designated pAR13438_2, which exhibited high transfer frequency without a significant fitness cost. In conclusion, CMY-178 is a novel CMY variant that mediates high-level resistance to ceftazidime-avibactam by enhancing the ability to hydrolyze ceftazidime and reducing the affinity for avibactam. Notably, *bla*_CMY-178_ could be transferred horizontally at high frequency without fitness costs.

**IMPORTANCE** Ceftazidime-avibactam is a novel β-lactam–β-lactamase inhibitor (BLBLI) combination with powerful activity against *Enterobacterales* isolates producing AmpC, such as CMY-like cephalosporinase. However, in recent years, CMY variants have been reported to confer ceftazidime-avibactam resistance. We reported a novel CMY variant, CMY-178, that confers high-level ceftazidime-avibactam resistance with potent transferability. Therefore, this resistance gene is a tremendous potential menace to public health and needs attention of clinicians.

## INTRODUCTION

Carbapenem-resistant *Enterobacterales* (CRE) members have become a global threat to public health (1). Controlling CRE infections is extremely challenging due to the limited number of effective treatments available. Avibactam is a novel β-lactamase inhibitor of class A, class C, and some class D beta-lactamases. Ceftazidime-avibactam exhibited potent *in vitro* activity against globally collected clinical isolates of *Enterobacterales*, including isolates that produce extended-spectrum beta-lactamases (ESBLs) and AmpC β-lactamases (2). In China, ceftazidime-avibactam has been approved to treat adults with complicated intra-abdominal infections (cIAIs), hospital-acquired pneumonia (HAP) (including ventilator-associated pneumonia [VAP]), and other infections caused by aerobic Gram-negative organisms, for which treatment options are limited. However, alarmingly, reports of resistance to ceftazidime-avibactam have increased gradually in recent years. The established mechanisms of resistance are linked mainly to targeted enzyme mutations, such as mutations in KPCs, CTX-Ms, and AmpCs (3). With the clinical application of ceftazidime-avibactam, an increasing number of studies have reported that KPC variants mediate ceftazidime-avibactam resistance. In fact, compared with other β-lactamase genes, CTX-M and AmpC genes are distributed more widely and deserve more attention.

CMY-2-like β-lactamases have become the most prevalent plasmid-borne *ampC* gene in Escherichia coli at the environmental interface between humans and animals (4). *bla*_CMY_s have been found in several types of plasmids, including IncC (formerly IncA/C_2_) and IncI1 (5). IncI1 plasmids have been isolated widely from animals and hospitalized patients worldwide, and their ability to be conjugatively transferred among different bacteria has received attention (6). Moreover, it has been reported that CMY combined with permeability alteration led to imipenem resistance in Klebsiella pneumoniae (7). Notably, the variants of CMY can decrease the sensitivity of *Enterobacterales* isolates to avibactam. *In vitro* selection experiments showed that Tyr150Ser and Asn346Ile substitutions in CMY-16 could enhance the ability of bacteria to resist avibactam and could increase the MIC of aztreonam-avibactam (8). However, CMY-mediated ceftazidime-avibactam resistance is found rarely in clinical isolates. Recently, it was reported that CMY-172 is responsible for ceftazidime-avibactam resistance in clinical carbapenem-resistant K. pneumoniae (CRKP) (9). Also, CMY-178, as described in this study, contributes a higher level of resistance to ceftazidime-avibactam than CMY-172.

In this study, we described *bla*_CMY-178_, a novel *bla*_CMY_ variant, that endowed multidrug-resistant (MDR) E. coli with high-level resistance to ceftazidime-avibactam. We used a recently reported ceftazidime-avibactam resistance gene, *bla*_CMY-172_, for comparison to explore why CMY-178 confers a higher level of resistance to ceftazidime-avibactam. Notably, the plasmid harboring *bla*_CMY-178_ exhibited excellent transferability, posing an enormous potential threat to public health.

## RESULTS

### Isolates and antibiotic susceptibility.

The clinical E. coli strain AR13438 was isolated from the ascites of a male patient with a complicated abdominal infection. Antimicrobial susceptibility testing showed that AR13438 was resistant to multiple cephalosporins and ceftazidime-avibactam but was susceptible to carbapenem and cefiderocol. AR13438 was resistant simultaneously to amikacin and levofloxacin (Table 1). Therefore, AR13438 was identified as MDR E. coli according to definitions established by Magiorakos et al. (10). Reference K. pneumoniae isolate KPCZA02 was a recently reported ceftazidime-avibactam-resistant strain mediated by *bla*_CMY-172_, which is also a CRKP strain (9).

**TABLE 1:** MICs and β-lactamase genes of the strains described in this study^*a*^

Strain	Description	β-Lactamase gene	MICs (μg/mL) of:														
			MEM	IMP	PIP	TZP	FOS	AMK	LEV	ATM	CTX	FEP	CXM	CAZ	CZA4	CZA8	CFDC
AR13438	Clinical E. coli isolate	*bla*_CMY-178_, *bla*_TEM-1_, *bla*_CTX-M-27_	0.125	1	>256	64/4	1	>256	32	32	>128	128	>1,024	2,048	64/4	32/8	0.25
KPCZA02	Clinical K. pneumoniae isolate	*bla*_KPC-2_, *bla*_CMY-172_, *bla*_TEM-1B_, *bla*_CTX-M-65_	128	64	>256	>256/4	>1,024	>256	32	>128	>128	>128	>1,024	2,048	128/4	64/8	1
E. coli DH5α			0.03	0.25	1	0.5/4	0.25	1	0.03	0.06	0.03	0.06	4	0.25	0.06/4	0.06/8	<0.03
E. coli DH5α-pCR2.1			0.03	0.25	1	0.5/4	0.25	1	0.03	0.12	0.06	0.12	4	0.25	0.12/4	0.12/8	<0.03
E. coli DH5α-pCR2.1-CMY-2	Transformant of the recombinant plasmid containing *bla*_CMY-2_	*bla*_CMY-2_	0.03	0.25	256	64/4	0.25	1	0.06	32	32	2	128	256	0.5/4		0.125
E. coli DH5α-pCR2.1-CMY-178	Transformant of the recombinant plasmid containing *bla*_CMY-178_	*bla*_CMY-178_	0.06	0.25	256	128/4	0.25	1	0.06	32	128	64	1,024	>2,048	64/4	32/8	1
E. coli DH5α-pCR2.1-CMY-172	Transformant of the recombinant plasmid containing *bla*_CMY-172_	*bla*_CMY-172_	0.06	0.25	256	128/4	0.25	2	0.03	8	32	16	64	512	16/4	2/8	1
E. coli J53			0.03	0.5	4	2/4	0.5	2	0.03	0.12	0.06	0.06	16	0.25	0.25/4	/^*b*^	/
E. coli J53-AR13438	Conjugant containing plasmid pAR13438_2	*bla*_CMY-178_	0.06	0.5	64	16/4	0.5	2	0.03	32	32	32	256	1024	32/4	/	/
E. coli J53-KPCZA02	Conjugant containing plasmid pKPCZA02_4	*bla*_CMY-172_	0.06	0.5	64	16/4	0.5	2	0.03	16	16	32	256	512	16/4	/	/

a MEM, meropenem; IMP, imipenem; PIP, piperacillin; TZP, piperacillin-tazobactam; FOS, fosfomycin; AMK, amikacin; LEV, levofloxacin; ATM, aztreonam; CTX, cefotaxime; FEP, cefepime; CXM, cefuroxime; CAZ, ceftazidime; CZA4, avibactam was tested at a fixed concentration of 4 μg/mL in combination with double dilutions of ceftazidime; CZA8, avibactam was tested at a fixed concentration of 8 μg/mL in combination with double dilutions of ceftazidime.

b /, no relevant measurements were taken.

AR13438 was identified as sequence type 457 (ST457) and serotype O25:H25. E. coli ST457 was identified as an extraintestinal pathogenic E. coli (ExPEC) strain and is an emerging ESBL-harboring lineage with reservoirs in wildlife and food-producing animals (11). The whole genome of AR13438 consisted of a 4,961,339-bp chromosome and two plasmids, named pAR13438_1 (124,392 bp) and pAR13438_2 (96,934 bp).

**FIG 1:**
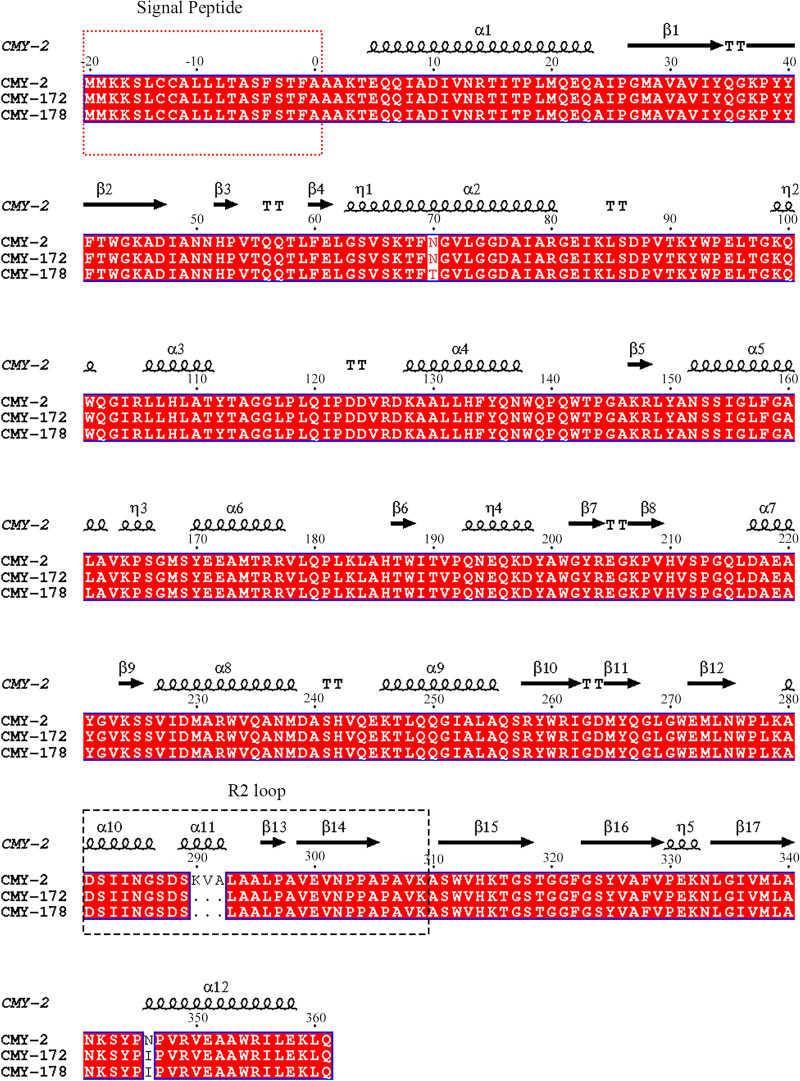
Amino acid sequence alignments of CMY-2, CMY-172, and CMY-178. The sequence alignments of CMYs were generated by Clustal Omega and ESPript 3.0. The secondary structure of CMY-2 is shown above the sequence, in which helices are squiggles, beta strands are arrows, and turns are TT letters. The sequence in the red dashed box is the signal peptide, and the sequence in the black dashed box is the R2 loop.

The acquired antibiotic resistance genes in AR13438 included *bla*_TEM-1_, *bla*_CTX-M-27_, *rmtB*, *aph(6)-Id*, *aph(3″)-Ib*, *aadA2*, *sul1 sul2*, *dfrA12*, *tet(A)*, *floR*, *mph(A)*, and a new variant of *bla*_CMY_ (designated *bla*_CMY-178_). Except for *bla*_CMY-178_, all antibiotic resistance genes listed above were located in pAR13438_1; *bla*_CMY-178_ was located in pAR13438_2. The alignment of CMY protein families showed that CMY-172 shared the highest amino acid identity with CMY-178. In both CMY-172 and CMY-178, three successive amino acids at positions 290 to 292 (K290_V291_A292del) were deleted and a nonsynonymous amino acid substitution was found at position 436 (Asn346Ile), in contrast to the CMY-2 protein. Furthermore, compared with CMY-172 and CMY-2, CMY-178 showed an additional nonsynonymous amino acid substitution at position 70 (Asn70Thr) (Fig. 1).

### Effects of CMY-178 on antibiotic susceptibility.

To discover the difference among *bla*_CMY_s in terms of the antimicrobial susceptibility profile, we cloned *bla*_CMY_s into pCR2.1 to construct pCR2.1_CMY-2, pCR2.1_CMY-172, and pCR2.1_CMY-178, which were introduced subsequently into E. coli DH5α. The expression levels of CMY-172 and CMY-178 decreased significantly compared with that of the wild type (CMY-2), but there was no significant difference between the expression levels of CMY-172 and CMY-178 (see Fig. S1 in the supplemental material). *bla*_CMY-2_ increased the cephalosporin MICs of DH5α by 16- to 512-fold and conferred piperacillin-tazobactam resistance to DH5α (Table 1). However, the CMY variants conferred stronger cephalosporin resistance, especially ceftazidime-avibactam resistance, to DH5α. Both CMY variants could hydrolyze β-lactam antibiotics other than carbapenems, while *bla*_CMY-178_ showed stronger resistance to cephalosporins than *bla*_CMY-172_ in DH5α (Table 1). Compared with *bla*_CMY-172_, *bla*_CMY-178_ increased the MICs of aztreonam, cefotaxime, and cefepime by 4-fold. The MICs of ceftazidime and cefuroxime increased over 4-fold. Notably, the presence of *bla*_CMY-172_ increased the ceftazidime-avibactam MIC from 0.12 μg/mL to 16 μg/mL, whereas *bla*_CMY-178_ increased the ceftazidime-avibactam MIC to 64 μg/mL.

### Kinetic parameters of CMYs.

Further experiments on enzyme kinetics were performed to define the altered kinetic properties impacted by Asn70Thr (Table 2). CMY-172 and CMY-178 exhibited the same *k*_cat_/*K_m_* values for nitrocefin, and thus, their hydrolysis rates of nitrocefin were almost the same. However, compared with CMY-2, the *k*_cat_/*K_m_* values of CMY-172 and CMY-178 decreased approximately 3-fold, which indicated that their rates of nitrocefin hydrolysis were weaker than that of CMY-2. The *K_i_* values of CMY-2, CMY-172, and CMY-178 against tazobactam were determined to be 0.76 ± 0.07, 2.12 ± 0.34, and 2.49 ± 0.51, respectively. Compared with CMY-2, CMY-172 and CMY-178 showed a higher *K_i_* for tazobactam, indicating that CMY-172 and CMY-178 exhibited a lower affinity for tazobactam. In comparison with CMY-172, CMY-178 exhibited a less than 2-fold increase in the *K_i_* value for tazobactam and a lower affinity for tazobactam. We believe that the second-order acylation rate constant (*k*_2_/*K_i_*) values of CMY-2, CMY-172, and CMY-178 for avibactam showed a similar trend to their *K_i_* values for tazobactam. However, we could not determine the *k*_2_/*K_i_* values of CMY variants for avibactam. Because CMY-172 and CMY-178 differ by a single amino acid substitution, we omitted the kinetics and used MICs to compare the inhibition effectiveness of avibactam. The expression levels of CMY-172 and CMY-178 were identical to those in E. coli DH5α. The MIC of ceftazidime-avibactam decreased from 16/4 μg/mL to 2/8 μg/mL when the concentration of avibactam increased from 4 μg/mL to 8 μg/mL in the transformant carrying *bla*_CMY-172_. However, the MIC of ceftazidime-avibactam decreased from 64/4 μg/mL to 32/8 μg/mL in the transformant carrying *bla*_CMY-178_. These inconsistent MIC changes supported that CMY-178 exhibited a greater resistant to avibactam than CMY-172.

**TABLE 2:** Kinetic parameters of CMY-2, CMY-172, and CMY-178

Molecule^*a*^	Data^*b*^ by variant											
	CMY-2				CMY-172				CMY-178			
	*K_m_* (μM)	*K*_cat_ (s^−1^)	*K*_cat_/*K_m_* (μM^−1^ s^−1^)	*K*_i_ (μM)	*K_m_* (μM)	*K*_cat_ (s^−1^)	*K*_cat_/*K_m_* (μM^−1^ s^−1^)	*K*_i_ (μM)	*K_m_* (μM)	*K*_cat_ (s^−1^)	*K*_cat_/*K_m_* (μM^−1^ s^−1^)	*K*_i_ (μM)
NCF	59.88 ± 2.59	732.18 ± 16.07	12.25 ± 0.60		12.02 ± 0.66	52.41 ± 2.65	4.36 ± 0.05		12.50 ± 0.31	55.81 ± 1.04	4.46 ± 0.08	
CAZ		NM^*c*^		(32.10 ± 2.10) × 10^−3^		NM		(1.06 ± 0.00) × 10^−3^		NM		(9.27 ± 0.29) × 10^−3^
TAZ				0.76 ± 0.07				2.12 ± 0.34				2.49 ± 0.51

a NCF, nitrocefin; CAZ, ceftazidime; TAZ, tazobactam.

b *K_m_*, *k*_cat_, and *K*_i_ values are shown as the means ± standard deviation from three independent experiments.

c NM, not measurable due to a low initial rate of hydrolysis.

**FIG 2:**
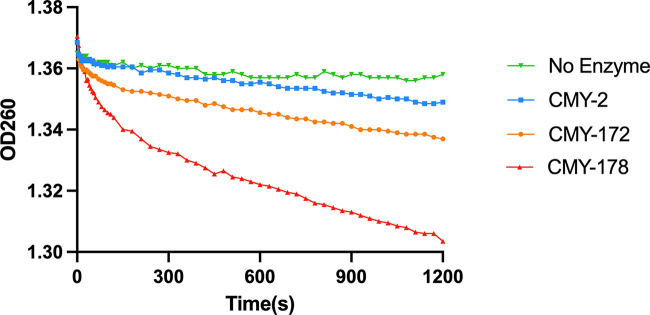
Progress curves of CMY-2 (blue), CMY-172 (orange), and CMY-178 (red) and no-enzyme control (green) for ceftazidime hydrolysis. All reactions were performed with 200 nM enzyme and 75 μM ceftazidime. Hydrolysis of ceftazidime results in a loss of absorbance at 260 nm.

Furthermore, the *K_m_* and *k*_cat_ values of CMY-2, CMY-172, and CMY-178 against ceftazidime could not be determined. Based on the *K_i_* values of ceftazidime, both CMY-172 and CMY-178 showed a higher affinity for ceftazidime than CMY-2; however, the *K_i_* value of CMY-178 for ceftazidime showed a substantial (~5-fold) increase compared with that of CMY-172. We used 200 nM enzymes and 75 μM ceftazidime to determine the hydrolysis curves, and the results supported that CMY-178 shows a stronger hydrolysis ability than CMY-172 (Fig. 2).

### Structural analysis of the ligand binding ability of CMYs.

To compare the binding modes of CMY-172 and CMY-178 at the molecular level, we prepared structural models. Avibactam formed hydrogen bonds with Ser64, Tyr150 Tyr221, and Ser31 of CMY-172 and with Ser64, Asn152, Gly318, and Arg346 of CMY-178. Ceftazidime formed hydrogen bonds with Ser64, Gln120, Tyr221, and Ser315 of CMY-172 and with Ser64, Gln120, Gly318, and Arg346 of CMY-178 (see Fig. S2 in the supplemental material). In addition, the aromatic carboxylic acid group of ceftazidime formed a salt bridge with Arg346 of CMY178, its thiazole ring formed π-π stacking with Tyr221 of CMY-178, and its pyridinium ion formed a π-cation with Tyr150 of CMY-172 and CMY-178 (Fig. S2). Other possible contact residues were Gly63, Leu119, Gln120, Glu272, Ser287, Thr313, Ser315, and Gly318 for both CMY variants. All these residues can stabilize ligand binding at the correct position for the reaction. Among them, the residues at positions 315, 318, and 346 influenced the affinity of lactamases mostly. For CMY-2, the ligands formed similar noncovalent bonds with similar residues. These residues and key contacts were consistent with ligand binding residues reported in the literature (12–15).

**FIG 3:**
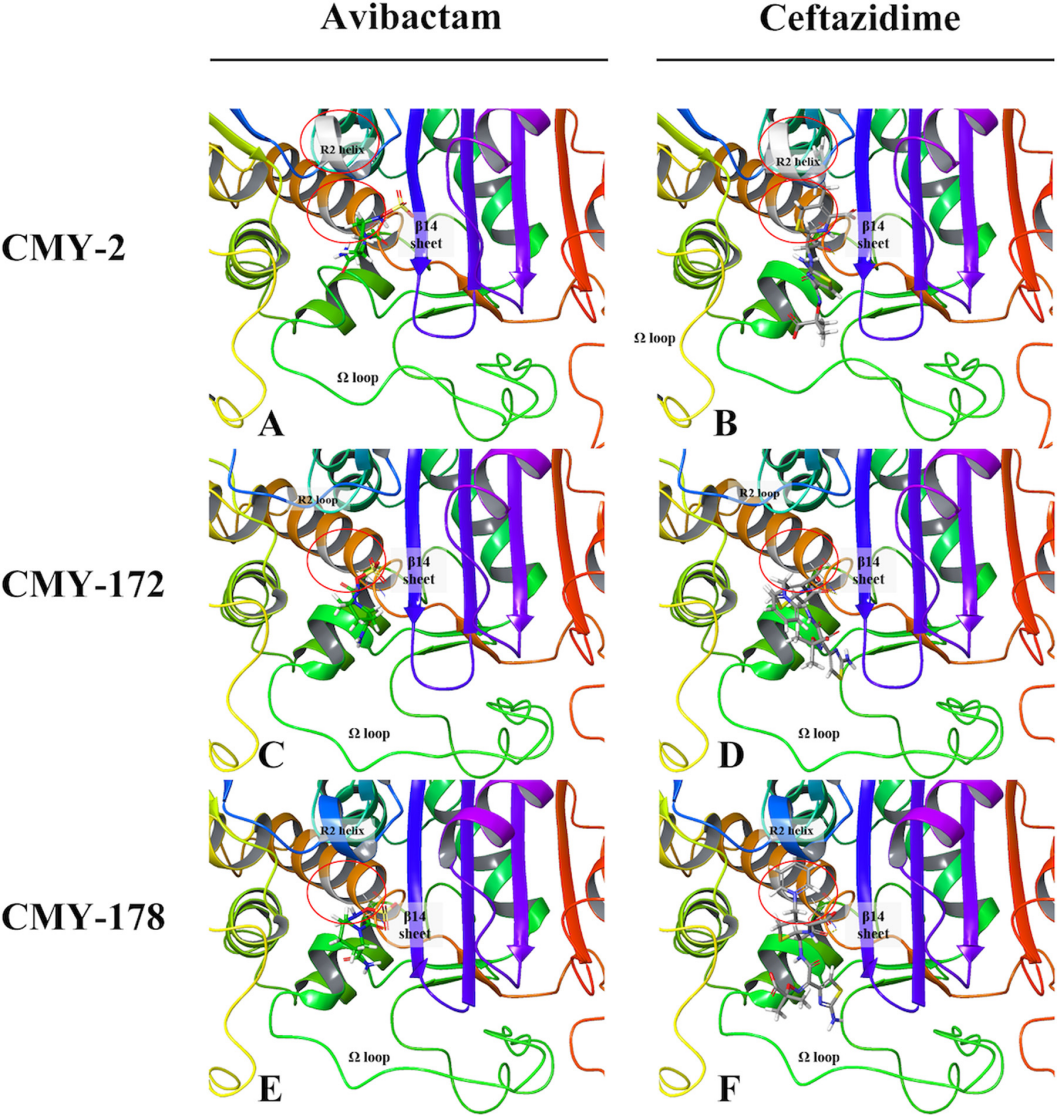
Molecular model of avibactam and ceftazidime binding with CMY-2, CMY-172, and CMY-178. (A and B) Denote avibactam and ceftazidime bound with CMY-2; the R2 helix is the silvery helix. (C and D) Denote avibactam and ceftazidime bound with CMY-172; the R2 loop is the blue loop. (E and F) Denote avibactam and ceftazidime bound with CMY-178; the R2 helix is the blue helix. The carbon of avibactam is colored in green, while the carbon of ceftazidime is colored in gray. Among them, mutation positions on the ribbons aligned to CMY-178 are colored in silver and marked by a red circle. The silver part of the brown helix marked with a red circle represents a mutation at position 70. The silver part of the R2 helix marked with a red circle represents a mutation at position 290 to 292.

**FIG 4:**
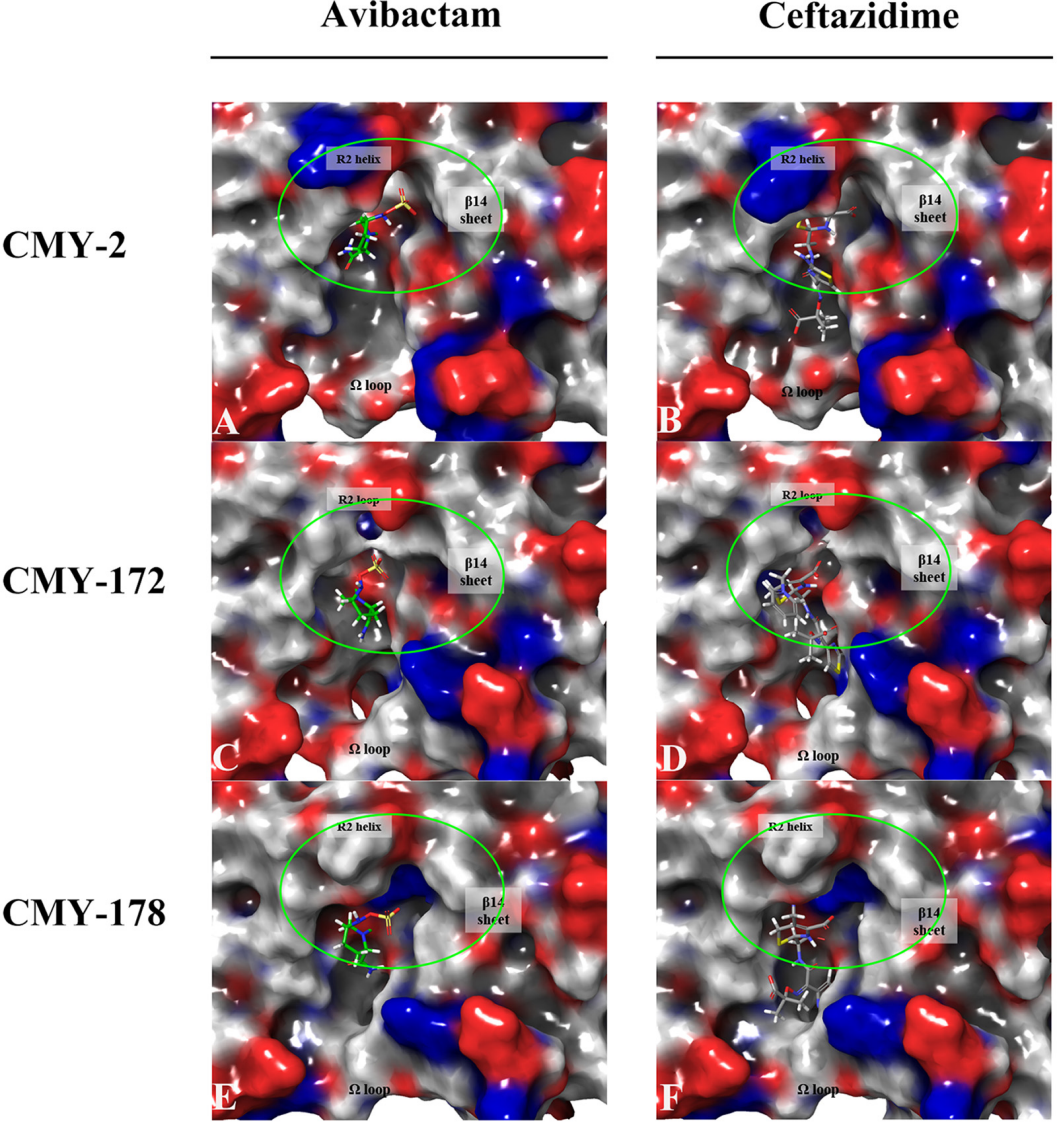
Residual charges on the binding surface of CMY-2, CMY-172, and CMY-178. (A and B) Denote avibactam and ceftazidime bound with CMY-2. (C and D) Denote avibactam and ceftazidime bound with CMY-172. (E and F) Denote avibactam and ceftazidime bound with CMY-178. Red refers to negative charges, and blue refers to positive charges. The carbon of avibactam is colored in green, while the carbon of ceftazidime is colored in gray. The sites marked by green circles are the main difference in the entrance of the ligand binding pocket among CMYs.

As shown in Fig. 3, the sulfate group in avibactam was always placed on the right side of the β14 sheet (His311-Phe319) and the R2 loop/helix (Ser287-Ala295), and the aliphatic carboxylic acid group and pyridinium ion in ceftazidime were always placed outward from the pocket and deep into the pocket, respectively. In addition, the pyridinium ion of ceftazidime always leaned beside the β14 sheet. The structure of tazobactam docking with CMYs was similar to that of avibactam (see Fig. S3 in the supplemental material). The R2 loop/helix in the upper-middle part of the plots and the Ω loop (Tyr203-Leu216) at the bottom of the plots were the key factors explaining the different ligand binding affinities and cephalosporin hydrolytic abilities of the two CMY variants (Fig. 3C to F). The R2 helix of CMY-178 bulged upward to shield the major entry site of the ligand binding pocket, whereas CMY-172 did not contain an R2 helix (Fig. 4C to F). However, the R2 loop of CMY-172 was negatively charged, while the R2 helix of CMY-178 was neutral and hydrophobic; thus, the requirements for charged ligand entries may be less stringent with CMY-178, and maintaining the ligands for reaction may be more difficult. As seen in Fig. 3C and F, the side chains of the β14 sheet and the Ω loop of CMY-178 swelled up to block the upper entry of the pocket. This structure differed from CMY-172, especially Leu290 of CMY-178, which was located at the same position as Ser289 of CMY-172. A comparison of the ligand binding pocket entrance revealed that the middle portion of the pocket lip widened but that the upper and lower portions of the pocket lip narrowed in CMY-178 (see Fig. S4 in the supplemental material). In addition, the residue charge distributions of the pocket entrances of the two CMY variants were different (Fig. 4D and F). Compared with CMY-172, CMY-178 contained more positive and neutral charges around the pocket entrance than CMY-172. All of the above-mentioned representations predicted that CMY-178 formed more noncovalent bonds with ligands and bound ligands more tightly, but it was more difficult for ligands to enter its pocket. We speculated that CMY-178 bound more closely to ceftazidime and helped it to form an acyl-enzyme complex more easily, so CMY-178 exhibited a stronger ability to hydrolyze ceftazidime than CMY-172.

### Conjugation and fitness cost of the *bla*_CMY-178_-carrying plasmid.

The conjugation experiment confirmed that *bla*_CMY-178_ could transfer its resistance to sodium-azide-resistant E. coli J53. S1-PFGE, and Southern blot analysis confirmed that the transmission of *bla*_CMY-178_ was achieved through plasmid conjugation (see Fig. S5 in the supplemental material). The presence of *bla*_CMY-178_ in transconjugants increased the MIC of ceftazidime-avibactam from 0.2 μg/mL to 32 μg/mL (Table 1). Based on three individual experiments, the mean value of the transfer frequencies was 4.20 × 10^−2^ transconjugants per recipient (see Table S1 in the supplemental material).

Plasmids introduce new antibiotic resistance genes into bacteria, but they also come with a cost, that is, the survivability of plasmid-carrying clones is reduced without the selection of plasmid-encoded traits. To evaluate the fitness cost of the CMY-178-carrying plasmid, the maximal growth rate, maximum optical density at 600 nm (OD_600_), and the duration of the lag phase were determined. The IncI1 plasmid containing *bla*_CMY-178_ (pAR13438_2) was transferred into E. coli J53 through conjugation to mimic the transmission of pAR13438_2 in nature. In Mueller-Hinton (MH) broth, J53 and J53-AR13438 exhibited similar growth curves, and their maximal growth rates, maximum OD_600_, and the duration of the lag phase were not significantly different (see Fig. S6 in the supplemental material).

### Genetic structure of the *bla*_CMY-178_-carrying plasmid.

*bla*_CMY-178_ was located in a plasmid named pAR13438_2, which belongs to the IncI1 type. *bla*_CMY-178_ was also the sole antibiotic resistance gene in pAR13438_2. As the conjugation experiment demonstrated, pAR13438_2 was a transferable plasmid that conferred ceftazidime-avibactam resistance. The plasmid pAR13438_2 contained a conjugal transfer region, resulting in the successful transmission of the plasmid, although the conjugation transfer system-related genes *traG* and *pliK* were interrupted by IS*4321* and IS*2* (16).

Analysis of the genetic environment of *bla*_CMY-178_ revealed the arrangement IS*1294-*ΔIS*Ecp1-bla*_CMY-178_*-blc-sugE-EcnR*. In the IncI1 plasmids carrying *bla*_CMY-2_, IS*1294-*ΔIS*Ecp1* and IS*1294-*IS*Ecp1* were the predominant upstream sequences of *bla*_CMY-2_, and the characteristic downstream sequences of *bla*_CMY-2_ were *blc* and *sugE* (17, 18). A similar fragment in pAR13438_2 was inserted into the colicin gene via the sequence GTTC that flanks the insertion sequence, as reported previously (5, 19–21). However, a part of the colicin gene *cia* was absent in pAR13438_2 (see Fig. S7 in the supplemental material). The preferred target sites at the 5′ end of IS*1294* were mainly, but not exclusively, the tetranucleotide sequence GTTC. We found that the sequence of pAR13438_2 was highly similar (coverage, 94%; identity, 99.96%) to that of pCE1628_I1 (GenBank accession no. MT468651.1), a plasmid of E. coli O11:H25 strain CE1628 (ST457) isolated from the feces of silver gulls in Australia. BLASTN showed another similar plasmid, pKPCZA02_4 (coverage, 93%; identity, 99.98%), from K. pneumoniae ST11 strain KPCZA02, which was isolated from a surgical intensive care unit and carries the ceftazidime-avibactam resistance gene *bla*_CMY-172_ (see Fig. S8 in the supplemental material). This finding suggested that the IncI1 plasmids carrying *bla*_CMY_ were relatively conserved and distributed worldwide.

## DISCUSSION

The current study indicated that a novel variant of a plasmid-encoded class C cephalosporinase CMY gene, *bla*_CMY-178_, mediated high-level resistance to ceftazidime-avibactam in MDR E. coli.

CMY-type β-lactamases are the most widely reported plasmid-carried AmpCs, and *bla*_CMY-2_ has been found worldwide in Salmonella spp. and E. coli (5). It has been reported that the Asn346Ile substitution in AmpC can increase the MIC of ceftazidime-avibactam for Pseudomonas aeruginosa (22). A previous study reported that a deletion mutation (G286_S287_D288) in AmpC generated an 8-fold decrease in susceptibility to ceftazidime in the clinical isolate E. coli HKY28 (23). Moreover, deletion at a similar position in AmpC (A294_P295) reduced the susceptibility of Enterobacter cloacae strain Ent385 to ceftazidime-avibactam and cefiderocol (24). CMY-172, which confers resistance to ceftazidime-avibactam, is a plasmid-encoded AmpC that was identified recently from a clinical CRKp strain. Researchers have hypothesized that the deletion mutation (K290_V291_A292del) and Asn346Ile substitution in CMY-172 coordinated to confer resistance to ceftazidime-avibactam (9). However, CMY-178 additionally contains an Asn70Thr substitution in contrast to CMY-172, and the resistance level of CMY-178 is higher than that of CMY-172. Moreover, the *K_i_* values of the two variants were not correlated with their MICs for ceftazidime. However, the ceftazidime hydrolysis curves of CMY variants confirmed that CMY-178 hydrolyzed ceftazidime faster. In fact, the *K_i_* value cannot be correlated with catalytic efficiency and hence with resistance levels. We hypothesized that the *k*_cat_ and *k*_cat_/*K_m_* values of CMY-178 for ceftazidime may have increased, so compared with CMY-172, CMY-178 could hydrolyze ceftazidime more quickly.

Due to the Asn70Thr mutation in CMY-178, the larger and more alkaline residue drives considerable changes in the adjacent β12 sheets (Leu293-Glu297). As a result, the R2 loop formed a short four-residue helix and the β14 sheet moved upward and away from the pocket, as shown in Fig. 3C and E. Thus, the top and right sides of the ligand binding pocket are very different between CMY-172 and CMY-178. As a result, the positive residue Ser315 in the lower portion of the β14 sheet of CMY-178 moved downward, resulting in the middle portion of the pocket lip widening but the upper and lower portions of the pocket lip narrowing (Fig. S4). In contrast to CMY-172, the positively charged His210 of the Ω loop in CMY-178 flipped out from the pocket, thereby obstructing ligands from entering the pocket. Ser64 of CMY-178 can form covalent bonds with the ligand more easily but more slowly. We speculated that the decreased affinity of CMY-178 for ceftazidime and avibactam occurred because the CMY-178 ligand binding pocket and its entrance narrowed and a more positive charge was present at the pocket entrance. However, since CMY-178 more easily forms an acyl-enzyme complex, its ability to hydrolyze ceftazidime is stronger than that of CMY-172.

AR13438 was identified as ST457 E. coli, which is an epidemic sequence type in wildlife, food-producing animals, and companion animals (25–29). The sequence of pAR13438_2 was highly similar to that of pCE1628_I1, a plasmid in E. coli O11:H25 strain CE1628 (ST457), isolated from the feces of silver gulls in Australia (GenBank accession no. MT468651.1). A similar plasmid was also detected in the E. coli isolate 4feg, which caused the death of a puppy in Italy (GenBank accession no. MN540570) (16). These plasmids were all identified as IncI1-type plasmids, which contain unique regions associated with plasmid replication, plasmid stability/maintenance, and transfer machinery (6). IncI1 plasmids have been isolated from multiple *Enterobacterales* strains originating from food animals and from clinically ill human patients, suggesting the possible transmission of IncI1 plasmids between human and animal populations (6). The *traEFG* genes likely form an operon that is not essential for conjugation to occur, and the function of the *pliK* gene remains unknown (6). This information may explain why the plasmid pAR13438_2 was still conjugable when the *traG* and *pliK* genes were interrupted. Moreover, a prior study showed that the acquisition of an IncI1 plasmid did not impose a significant fitness cost on the host bacteria (30).

However, due to the transferability of the IncI1 plasmid with low fitness cost and the rolling circle transposition of IS*1294*, the transfer of ceftazidime-avibactam resistance mediated by *bla*_CMY-178_ warrants attention.

In conclusion, we reported here a novel *bla*_CMY-178_ gene that confers high-level ceftazidime-avibactam resistance in MDR E. coli and assessed the biochemical properties of CMY-178. Through protein structural analysis, we found that the Asn70Thr substitution in CMY-178 caused a decrease in its affinity but an increase in its hydrolytic capacity. Furthermore, *bla*_CMY-178_ is associated with the *IS*1294-mediated element and IncI1 plasmid with a high conjugation frequency, which indicates a potential risk of horizontal transmission. Therefore, clinicians must focus on novel resistance genes, and genetic surveillance is necessary.

## MATERIALS AND METHODS

### Bacterial isolates.

E. coli isolate AR13428 was collected in 2020 in a tertiary hospital in China. The reference K. pneumoniae isolate KPCZA02 carrying the *bla*_CMY-172_ gene was provided by Yiqi Fu (9). Species were identified using matrix-assisted laser desorption ionization–time of flight mass spectrometry (MALDI-TOF MS) (Bruker Daltonics, Bremen, Germany).

### Whole-genome sequencing.

The genomic DNA of E. coli isolate AR13428 was extracted by using a QIAamp DNA minikit (Qiagen, New York) and subjected to Illumina paired-end sequencing (Illumina Inc., San Diego, CA).To explore the complete structure of the plasmid, E. coli isolate AR13428 was sequenced using long-read Nanopore sequencing (Oxford Nanopore Technologies, Oxford, UK).The reads of Illumina sequencing were assembled to contigs using Shovill 0.9.0 (https://github.com/tseemann/shovill), and the hybrid assembly of Illumina and Nanopore reads was performed by Unicycler v0.4.8 (https://github.com/rrwick/Unicycler). Antibiotic resistance genes (ARGs) were recognized using ABRicate v1.0.1 (https://github.com/tseemann/abricate) or BLAST (https://blast.ncbi.nlm.nih.gov/Blast.cgi) based on the NCBI database. Plasmid incompatibility types were identified with the Center for Genomic Epidemiology guidelines (https://cge.food.dtu.dk/services/PlasmidFinder/). Multilocus sequence typing (MLST) was performed with mlst v2.19.0 (https://github.com/tseemann/mlst) based on Achtman scheme. Comparisons of plasmid sequences were performed using Proksee (https://proksee.ca/). The conjugative regions of the plasmid were analyzed by oriTfinder (https://tool-mml.sjtu.edu.cn/oriTfinder/oriTfinder.html). The whole-genome sequences of KPCZA02 were downloaded from the GenBank database (accession no. CP058226 to no. CP058230).The amino acid sequence alignment of CMYs was generated by Clustal Omega and ESPript 3.0 (31, 32). The signal peptides were sorted by minus values, and the mature protein was numbered according to the consensus class C numbering system of Mack et al. (33).

### Antimicrobial susceptibility testing.

Antimicrobial susceptibility testing was performed and interpreted according to the Clinical and Laboratory Standards Institute (CLSI) guidelines and breakpoints (M100, 31st edition) by the agar dilution method. E. coli ATCC 25922 and K. pneumoniae ATCC 700603 served as control strains.

The antibiotics we used included meropenem (Hanhui Pharmaceutical Co., Ltd., China), imipenem (Merck Sharp & Dohme Corp., USA), cefepime (Jiangsu Hengrui Pharmaceutical Co., Ltd., China), piperacillin (Suzhou Erye Pharmaceutical Co., Ltd., China), ceftazidime (Guangdong Jincheng Jinsu Pharmaceutical Co., Ltd., China), tazobactam (Meilunbio, China), avibactam (MedChemExpress, USA), aztreonam (Sigma-Aldrich, USA), amikacin (Meilunbio), cefuroxime (Esseti Farmaceutici S.r.l., Italy), fosfomycin (Harbin Pharmaceutical Group Co., Ltd. China), levofloxacin (Daiichi Sankyo Company Limited, Beijing, China), and cefotaxime (North China Pharmaceutical Hebei Huamin Pharmaceutical Co., Ltd., China).

### Gene cloning.

The *bla*_CMY_ cloning was performed using a TA cloning kit (Invitrogen, Shanghai, China). The *bla*_CMY-178_ gene combined with its predicted promoter was amplified from isolate AR13438. Then, the *bla*_CMY-178_ gene was cloned into the linearized vector pCR2.1 using T4 DNA ligase (Invitrogen). The *bla*_CMY-172_ gene was cloned from KPCZA02 with the same method. The resulting plasmids were chemically transformed into E. coli DH5α. The positive transformants were identified by blue-white screening in MH agar containing 50 μg/mL kanamycin and were further confirmed by Sanger sequencing. The pCR2.1 blank vector was also transformed into E. coli DH5α as a control. The primers used for cloning were CMY-F and CMY-R (see Table S2 in the supplemental material). Relative CMY expression was measured by reverse transcription-quantitative PCR as described previously (34). Specific quantitative PCR (qPCR) primers (q-recA-F/R and q-CMY-F/R) were designed (Table S2).

### Plasmid transfer experiments.

Conjugation experiments were performed by filter mating using sodium-azide-resistant E. coli J53 as the recipient, as described previously. Transconjugants were selected with MH agar containing 100 μg/mL sodium azide and 4 μg/mL ceftazidime-avibactam; furthermore, transconjugants were named J53-AR13438 and J53-KPCZA02, respectively. We compared genomes of AR13438 and J53 to select a unique gene (*gtrA*) to J53. The *bla*_CMY-178_-postive transconjugants were confirmed by PCR and MALDI-TOF MS (see Fig. S9 in the supplemental material). The primers used for PCR were gtrA-F, gtrA-R, CMY-F, and CMY-R (Table S1). The transfer frequencies were calculated as the number of transconjugants (CFU/mL) divided by the number of recipients (CFU/mL). Donors and recipients were mixed at 1:1 for the transfer frequency experiment. S1-PFGE and Southern blotting were performed to define the location of *bla*_CMY-178_ and confirm the transmission of the plasmid as described previously (35). The MIC profiles of transconjugants were also determined by the agar dilution method.

### Growth rate determination.

To evaluate the fitness cost of the strain carrying the *bla*_CMY_-harboring plasmids pAR13438_2 and pKPCZA02_4, three independent cultures of J53, J53-AR13438, and J53-KPCZA02 were grown overnight in MH broth at 37°C. Then each culture was diluted 1:1,000 in MH broth (with or without antibiotic) and added into a flat-bottom honeycomb 100-well plate. The OD_600_ of triplicates was measured every 5 min for 20 h using a Bioscreen C MBR machine (Oy Growth Curves Ab Ltd., Finland). The maximal growth rate was estimated based on OD_600_ curves using an R script, as described previously (36). Statistical analysis was performed with a *t* test in GraphPad Prism 9.0.

### Purification of CMYs and measurement of enzyme kinetics.

*bla*_CMY_s (including *bla*_CMY-2_, *bla*_CMY-172_, and *bla*_CMY-178_) were amplified without a signal peptide and cloned into the pET28a, which led to the introduction of an N-terminal His_8_-Tag.The recombinant plasmids were transformed into E. coli BL21(DE3) and selected using Sanger sequencing. Lysogenic broth containing 50 μg/mL kanamycin was inoculated with transformants and grown to reach an OD_600_ of 0.6. A final concentration of 1 mM isopropyl-β-d-thiogalactopyranoside (IPTG) was added into the resulting culture, which was shaken at 18°C overnight. The culture broth was centrifuged to harvest bacteria. The pellet was resuspended in lysis buffer (50 mM Tris-HCl and 300 mM NaCl (pH 7.5)) and disrupted using a JN-02C French press (JNBIO, Guangzhou, China) at 1,100 bar. Then, the cell debris was removed by centrifugation at 11,000 rpm for 40 min at 4°C. The supernatant was added to an Ni column, and the absorbed protein was eluted by the eluent buffer (lysis buffer with 300 mM imidazole). SDS-PAGE confirmed the protein purity of the eluent (>95%), and the sample was loaded onto a pre-equilibrated Superdex 200 column to eliminate imidazole.

Enzymatic activities were measured using a D8 UV-visible spectrophotometer (Rumqee, Shanghai, China) in phosphate-buffered saline (PBS; pH 7.2) at room temperature. Substrates tested included nitrocefin, ceftazidime, and avibactam. The extinction coefficient and wavelength of nitrocefin are 17,400 M^−1^ · cm^−1^ and 482 nm, respectively. The *K_i_* values of ceftazidime were determined by performing direct competition assays with 100 μM nitrocefin ([S]) as substrates and a 2-min incubation at room temperature. The initial reaction velocities (*v*) were measured and *K_i_* values were calculated by fitting data into equation 1 with GraphPad Prism v9.0.0 (37).

(1) $$v=\frac{V_{\max}\left[\text{S}\right]}{\left[\text{S}\right]+K_{\text{m NCF }}(1+\frac{\left[\text{I}\right]}{K_{\text{i}}})}$$

Progress curves of CMY variants for ceftazidime hydrolysis were generated by measuring absorbance at 260 nm with 200 nM enzymes and 75 μM ceftazidime (38).

### Structure modeling and molecular docking.

After PSI-BLAST (39) and MUSCLE alignment (40), the crystal structures of class C β-lactamases CMY-136 (PDB 6G9T) retrieved from the RCSB Protein Data Bank (PDB) were applied as the structure template of CMY-172 and CMY-178. We constructed a homology model filling the template junctions and deletions using the “prime homology modeling” module in Schrödinger Release 2018-1 separately (41). In addition, the citrate-bound crystal structure of CMY-2 (PDB 1ZC2) was used as the initial structure for modeling. We renumbered amino acid residues based on the primary structure of CMY-172 and CMY-178. After protein modeling was complete, the protein structure at the neutral pH was preprocessed and refined with Protein Preparation Wizard (42). We used the OPLS3e force field to restrain energy minimization (43). Afterward, protein systems were further reviewed to ensure no steric clashes or other deviations. Additionally, ligands were prepared before docking.

To localize the exact binding pocket, we set Ser64 as the centroid of the grid box center and H-bond constraint atoms and applied the “induced fit docking protocol” in Schrödinger (44) to dock the ligands and CMY proteins. According to the previous high-resolution structure analysis, we filtered out correct docking poses in which Ser64 and Tyr150 have hydrogen bonds with the aromatic carbonyl group of ligands (12). Then we ranked the correct poses and selected the top pose as the initial structure of covalent docking.

The “covalent docking protocol” in Schrödinger (45) was used to identify the catalyzing conformation. The β-lactam addition and self-defined diazabicyclooctane addition were chosen as the reaction types separately; the refinement minimization radius was set to 5.0 Å.

### Ethics statement.

Approval was obtained from the Ethics Committee of Sir Run Run Shaw Hospital (approval/reference number: 20200831-36).

### Data availability.

The nucleotide sequences in this study have been submitted to the GenBank database under accession no. OK554431 (the *bla*_CMY-178_ gene) and CP097170 to CP097172 (chromosome and two plasmids of AR13438).
