# Supplementary material for: A Novel CMY Variant Confers Transferable High-Level Resistance to Ceftazidime-Avibactam in Multidrug-Resistant Escherichia coli
Source: Microbiol Spectr. 2023 Feb 14;11(2):e03349-22. doi: 10.1128/spectrum.03349-22 (PMC10100771; doi:10.1128/spectrum.03349-22)
Supplement: Supplemental file 1 — Supplemental material. Download spectrum.03349-22-s0001.pdf, PDF file, 8.9 MB [file spectrum.03349-22-s0001.pdf]

Legends

Table S1. Transfer frequency of pAR3438_2 from AR13438 to J53

Table S2. Primers used in this study

Figure S1. Expression levels of different CMY variants in *E. coli* DH5α.
ns, no significance; ****, P<0.0001

Figure S2. Avibactam and ceftazidime modeled in the active site of CMY-2, CMY-172 and CMY-178 as an acylated complex. (A) and (B) denote avibactam and ceftazidime bound with CMY-2 separately, (C) and (D) denote avibactam and ceftazidime bound with CMY-172 separately, while (E) and (F) denote avibactam and ceftazidime bound with CMY-178 separately. The carbon of avibactam is colored in green, while carbon of ceftazidime is colored in gray. Hydrogen bonds are expressed as yellow dotted lines, aromatic hydrogen bonds are expressed as cyan dotted lines, salt bridges are expressed as magenta dotted lines, π-π interactions are expressed as azure dotted lines and π-cation interactions are expressed as green dotted lines.

Figure S3. Binding mode analysis of tazobactam bound to CMY-2, CMY-172 and CMY-178 as an acylated complex. (A) and (B) denote tazobactam bound to CMY-2 separately, (C) and (D) denote tazobactam bound to CMY-172 separately, while (E) and (F) denote tazobactam bound to CMY-178 separately. (A), (C) and (E) shows the ribbon representation of lactamase, (B), (D) and (F) shows the binding surface of lactamase with residue charges respectively, where red refers to negative charges and blue refers to positive charges. Among them, mutation positions on the ribbons aligned to CMY-178 are colored in platinum. The carbon of tazobactam is colored in cyan.

Figure S4. Comparison of the ligand-binding pocket entrance of CMY variants. (A) CMY variants bind with avibactam. (B) CMY variants bind with ceftazidime. CMY-172 is colored in purple, CMY-178 is colored in green.

Figure S5. Analysis of the location of blaCMY-178 by S1-PFGE (left part) and Southern Blot (right part).M, *Salmonella Braenderup* H9812 digested by *Xba*I for marker. Lane 1-2, blaCMY-178 carrying E. coli strain AR13438; lane 3-4, E. coli J53 transconjugant containing blaCMY-178 carrying plasmid

Figure S6. Fitness cost of pAR13438_2. J53-AR13438: transconjugants of AR13438. (A) Growth curve of J53 and J53-AR13438. (B) Relative growth rate of J53 and J53-AR13438. Maximum growth rate of J53 was normalized to 1.0 and ratio of transconjugants to J53 was relative growth rate. (C) Maximum OD_{600} of J53 and J53-AR13438. (D) Relative Lag time of J53 and J53-AR13438. The duration of the lag phase of J53 was normalized to 1.0 and ratio of transconjugants to J53 was relative Lag time. ns, no significance.

Figure S7. Genetic environment of *bla*$_{CMY-178}$ in plasmid pAR13438_2 and comparison with other plasmids pKPCZA02_4 and pCE1628_I1. Target sequences of insertion (GTTC and CTTG) were labeled. *blc*, outer membrane lipoprotein gene; *sugE*, quaternary ammonium compound efflux SMR transporter gene; *ecnR*, LuxR family transcriptional regulator gene.

Figure S8. Comparison of *bla*$_{CMY-178}$ carrying plasmid with other plasmids using Proksee. The location of conjugal transfer genes, plasmid replication protein gene, *bla*$_{CMY-178}$ and IS elements are labeled. GenBank accession No. of the plasmids: pCE1628_I1, MT468651.1; pIV_IncI1, MN540570.1; pKPCZA02_4, CP058230; pAR13438_2, CP097172.

Figure S9. Agarose gel electrophoresis of PCR-amplified products from J53, AR13438 and transconjugants J53-AR13438. (A) Lane M, 2,000 bp DNA marker; lane 1 and lane 2, AR13438(amplified for blaCMY-178 gene); lane 3, J53(amplified for blaCMY-178 gene); lane 4 and lane 5, transconjugant J53-AR13438(amplified for blaCMY-178 gene). (B) Lane M, 2,000 bp DNA marker; lane 1 and lane 2, AR13438(amplified for J53-specific gtrA gene); lane 3=, J53(amplified for J53-specific gtrA gene); lane 4 and lane 5, transconjugant J53-AR13438(amplified for J53-specific gtrA gene).

.

TableS1.Transfer frequency of pAR3438_2 from AR13438 to J53

	Experiment			
	1	2	3	Mean
#Recipients(R)	5.00×10^{9}	5.40×10^{9}	5.70×10^{9}	
#Transconjugants (TC)	2.22×10^{8}	2.22×10^{8}	2.30×10^{8}	
Conjugation frequency (TC/R)	4.44×10^{-2}	4.11×10^{-2}	4.04×10^{-2}	$(4.20\pm 0.18)\times10^{-2}$

Table S2. Primers used in this study

Genes	Primer Sequence (5'→3')
bla$_{CMY-178}$	CMY-F: GTAAATAATGTTACAATGTGTGAGAAGCA
	CMY-R: GGAAAAAAAGAGAAAGAAAG
gtrA	gtrA-F: TCATTTCGCATCCCTAAAGA
	gtrA-R: ATGTTAAAGCTATTTGCAAAG
recA	q-recA-F: GTAAAGGCTCCATCATGCGC
	q-recA-R: AGATTTCGACGATACGGCCC
bla$_{CMY}$	q-CMY-F: TCGCCAATAACCACCCAGTC
	q-CMY-R: GACCGGATCGCTGAGCTTAA

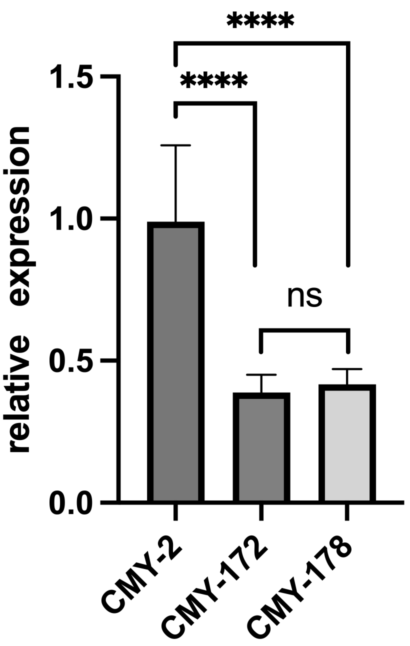

Figure S1. Expression levels of different CMY variants in *E. coli* DH5α.
ns, no significance; ****, P<0.0001

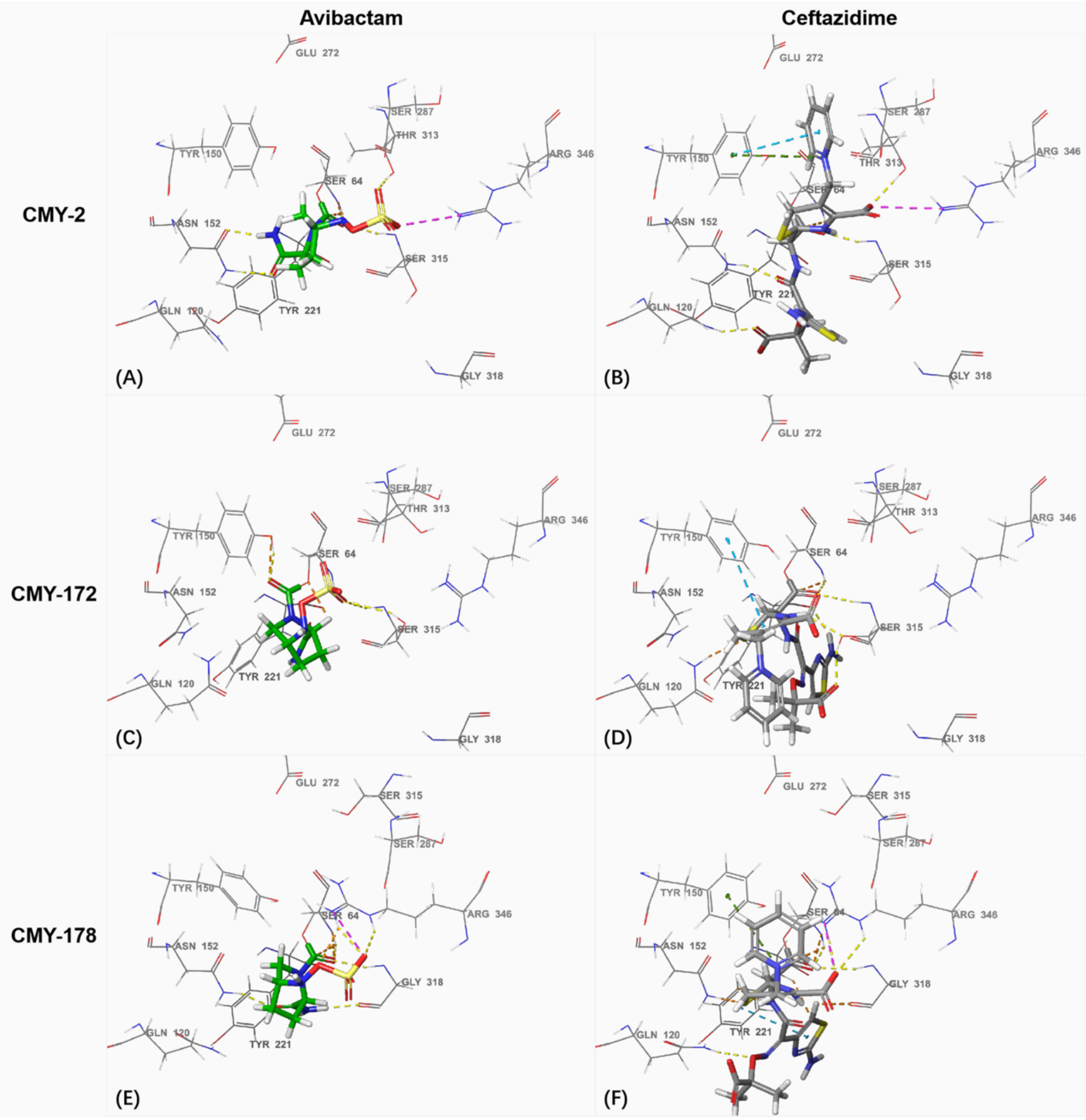

Figure S2. Avibactam and ceftazidime modeled in the active site of CMY-2, CMY-172 and CMY-178 as an acylated complex. (A) and (B) denote avibactam and ceftazidime bound with CMY-2 separately, (C) and (D) denote avibactam and ceftazidime bound with CMY-172 separately, while (E) and (F) denote avibactam and ceftazidime bound with CMY-178 separately. The carbon of avibactam is colored in green, while carbon of ceftazidime is colored in gray. Hydrogen bonds are expressed as yellow dotted lines, aromatic hydrogen bonds are expressed as cyan dotted lines, salt bridges are expressed as magenta dotted lines, π-π interactions are expressed as azure dotted lines and π-cation interactions are expressed as green dotted lines.

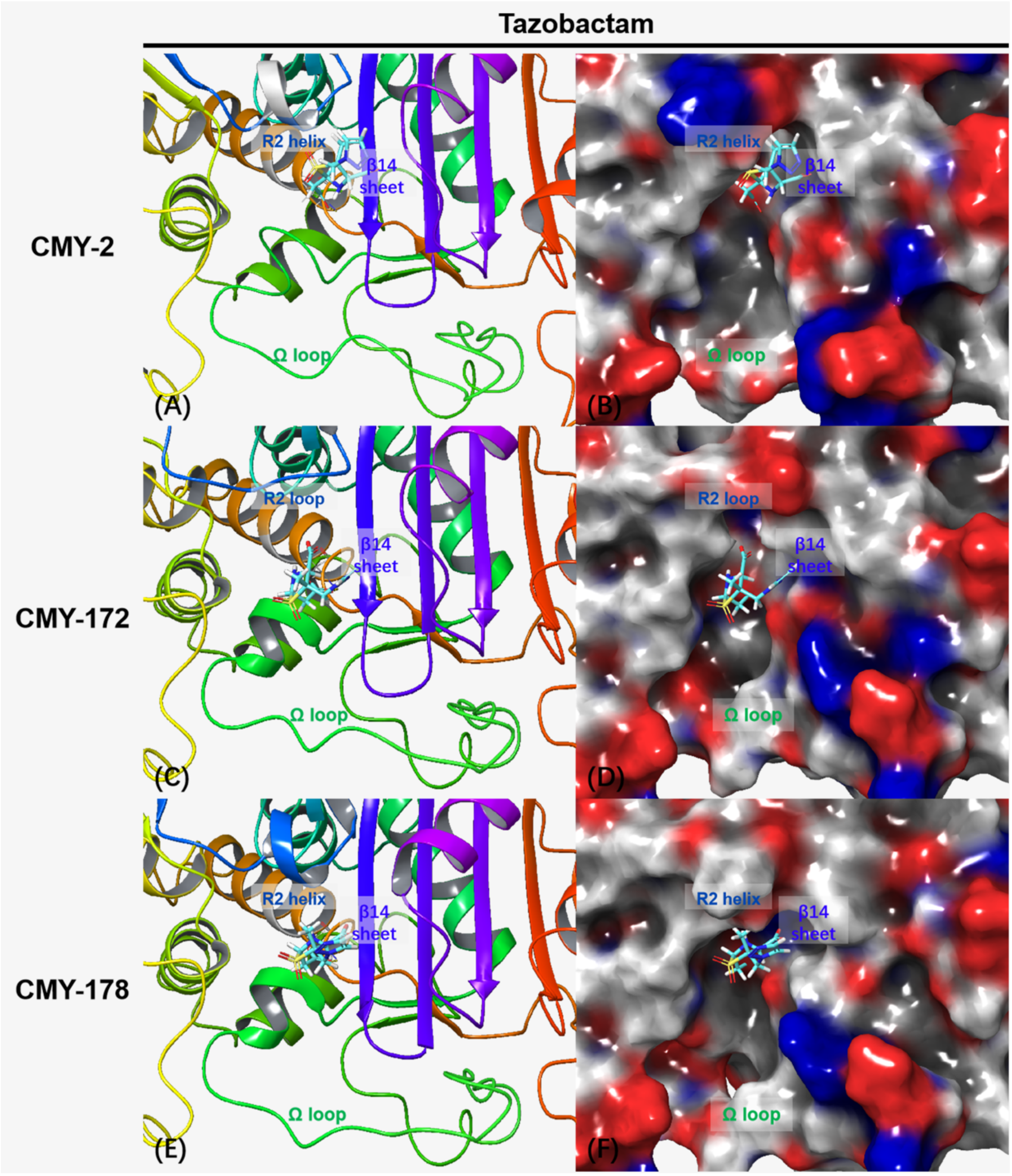

Figure S3. Binding mode analysis of tazobactam bound to CMY-2, CMY-172 and CMY-178 as an acylated complex. (A) and (B) denote tazobactam bound to CMY-2 separately, (C) and (D) denote tazobactam bound to CMY-172 separately, while (E) and (F) denote tazobactam bound to CMY-178 separately. (A), (C) and (E) shows the ribbon representation of lactamase, (B), (D) and (F) shows the binding surface of lactamase with residue charges respectively, where red refers to negative charges and blue refers to positive charges. Among them, mutation positions on the ribbons aligned to CMY-178 are colored in platinum. The carbon of tazobactam is colored in cyan.

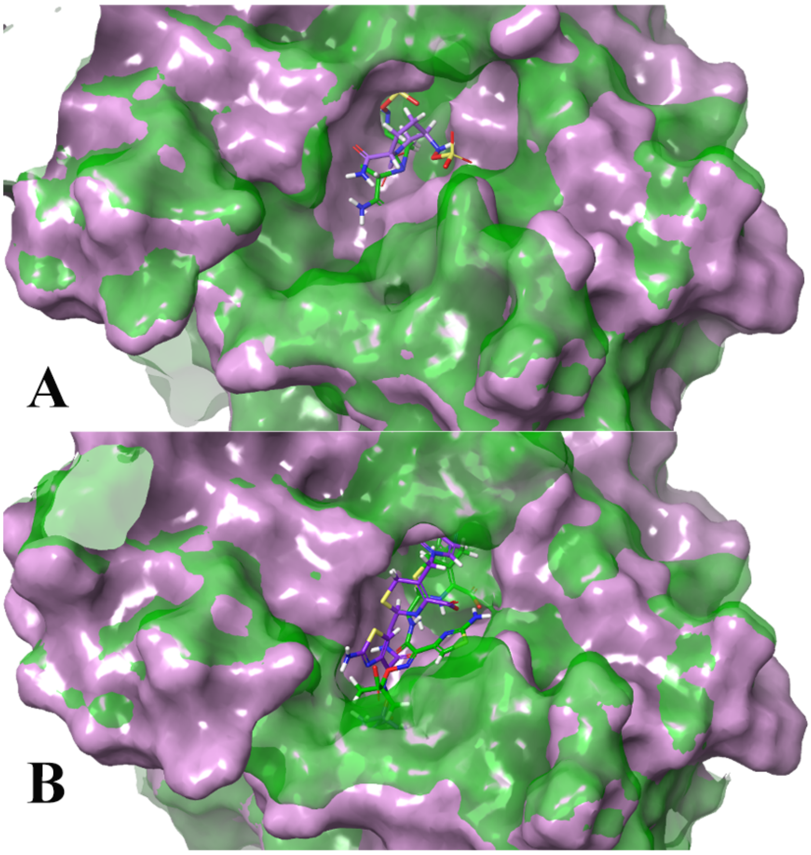

Figure S4. Comparison of the ligand-binding pocket entrance of CMY variants. (A) CMY variants bind with avibactam. (B) CMY variants bind with ceftazidime. CMY-172 is colored in purple, CMY-178 is colored in green.

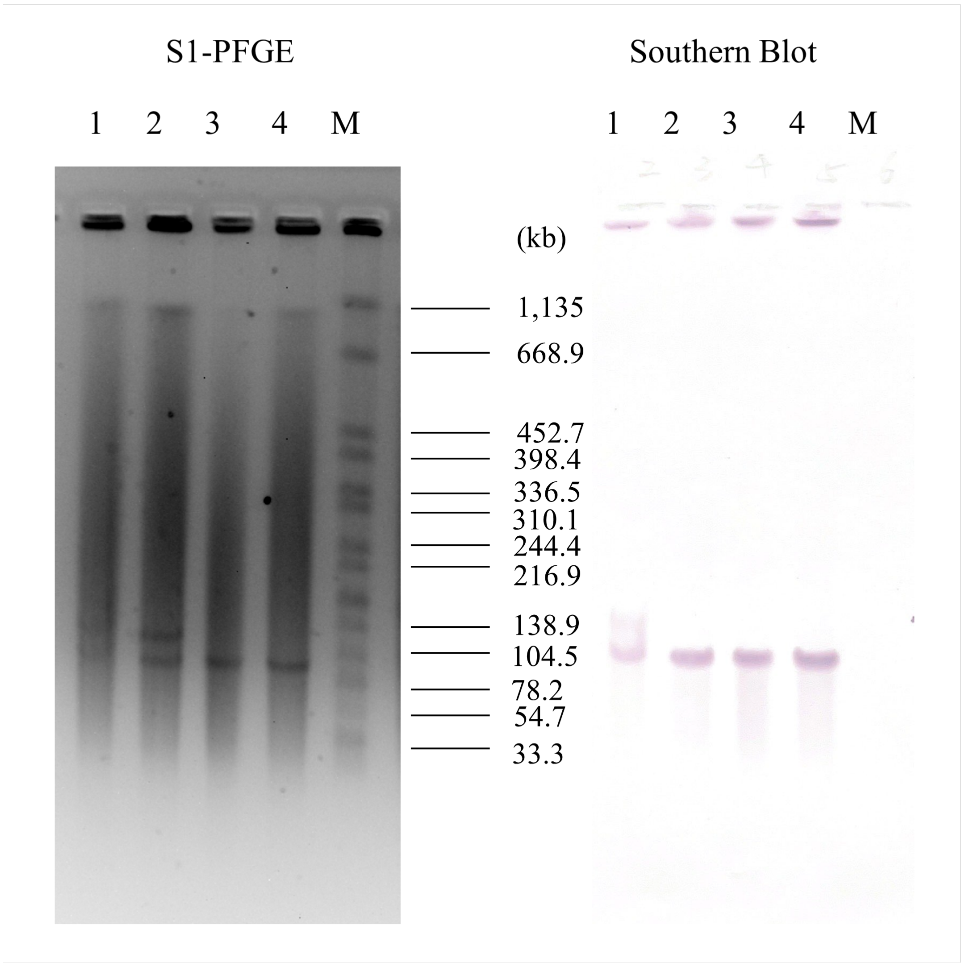

Figure S5. Analysis of the location of blaCMY-178 by S1-PFGE (left part) and Southern Blot (right part).M, *Salmonella Braenderup* H9812 digested by *Xba*I for marker. Lane 1-2, blaCMY-178 carrying E. coli strain AR13438; lane 3-4, E. coli J53 transconjugant containing blaCMY-178 carrying plasmid

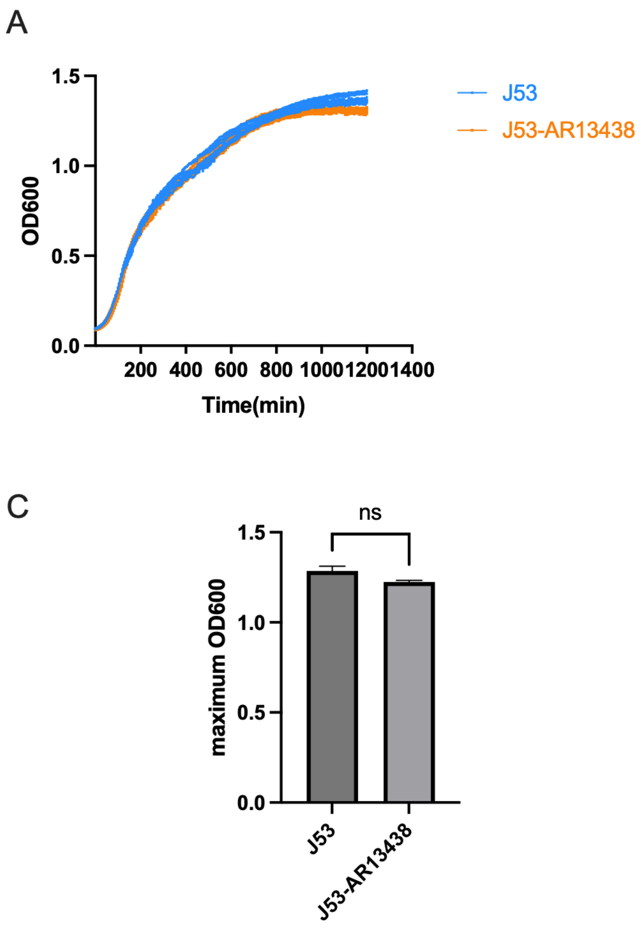

Figure S6. Fitness cost of pAR13438_2. J53-AR13438: transconjugants of AR13438. (A) Growth curve of J53 and J53-AR13438. (B) Relative growth rate of J53 and J53-AR13438. Maximum growth rate of J53 was normalized to 1.0 and ratio of transconjugants to J53 was relative growth rate. (C) Maximum OD_{600} of J53 and J53-AR13438. (D) Relative Lag time of J53 and J53-AR13438. The duration of the lag phase of J53 was normalized to 1.0 and ratio of transconjugants to J53 was relative Lag time. ns, no significance.

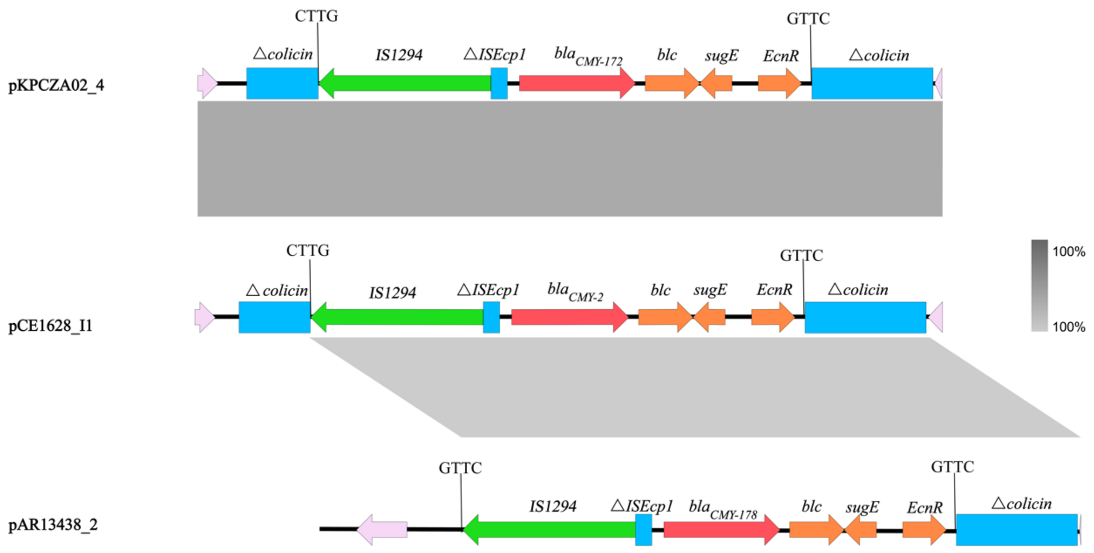

Figure S7. Genetic environment of *bla*$_{CMY-178}$ in plasmid pAR13438_2 and comparison with other plasmids pKPCZA02_4 and pCE1628_I1. Target sequences of insertion (GTTC and CTTG) were labeled. *blc*, outer membrane lipoprotein gene; *sugE*, quaternary ammonium compound efflux SMR transporter gene; *ecnR*, LuxR family transcriptional regulator gene.

.

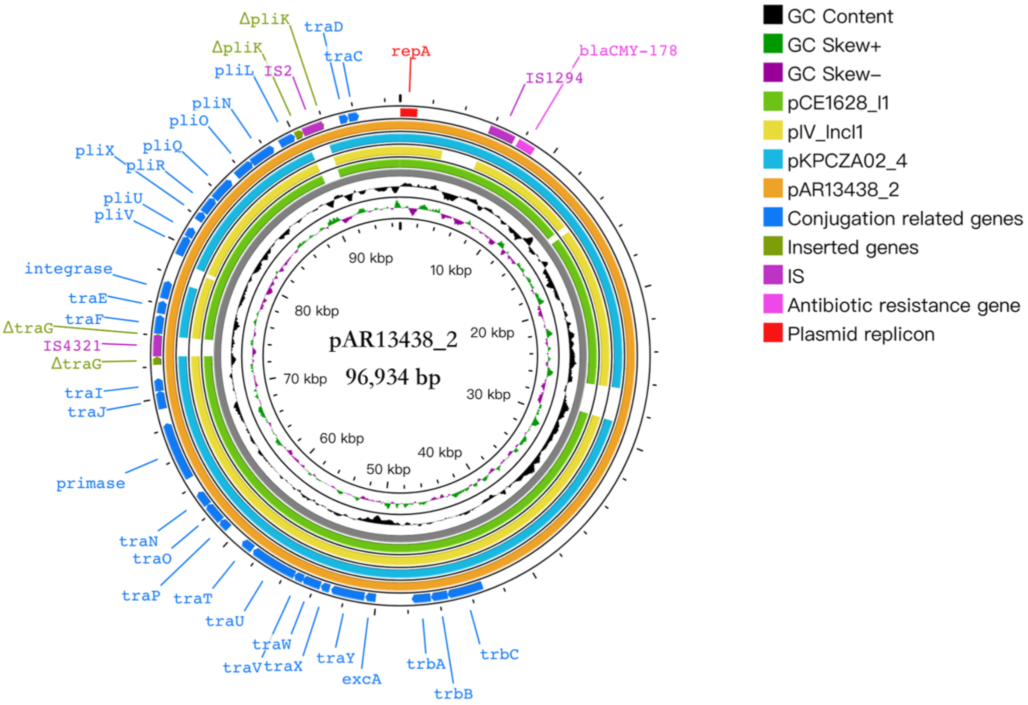

Figure S8. Comparison of *bla*$_{\text{CMY-178}}$ carrying plasmid with other plasmids using Proksee. The location of conjugal transfer genes, plasmid replication protein gene, *bla*$_{\text{CMY-178}}$ and IS elements are labeled. GenBank accession No. of the plasmids: pCE1628_I1, MT468651.1; pIV_IncI1, MN540570.1; pKPCZA02_4, CP058230; pAR13438_2, CP097172.

.

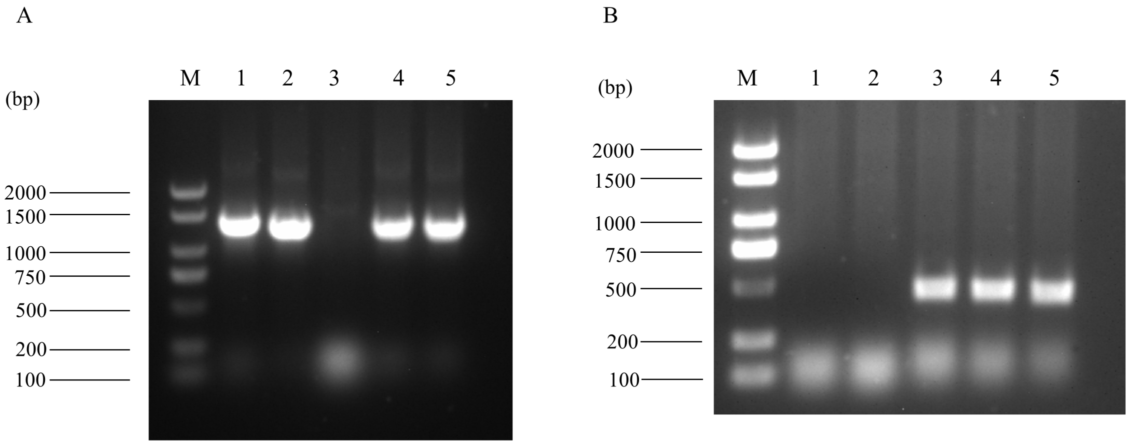

Figure S9. Agarose gel electrophoresis of PCR-amplified products from J53, AR13438 and transconjugants J53-AR13438. (A) Lane M, 2,000 bp DNA marker; lane 1 and lane 2, AR13438(amplified for blaCMY-178 gene); lane 3, J53(amplified for blaCMY-178 gene); lane 4 and lane 5, transconjugant J53-AR13438(amplified for blaCMY-178 gene). (B) Lane M, 2,000 bp DNA marker; lane 1 and lane 2, AR13438(amplified for J53-specific gtrA gene); lane 3=, J53(amplified for J53-specific gtrA gene); lane 4 and lane 5, transconjugant J53-AR13438(amplified for J53-specific gtrA gene).
